# Controlling for Confounding Effects in Single Cell RNA Sequencing Studies Using both Control and Target Genes

**DOI:** 10.1038/s41598-017-13665-w

**Published:** 2017-10-19

**Authors:** Mengjie Chen, Xiang Zhou

**Affiliations:** 1 Department of Medicine, University of Chicago, Chicago, IL 60637 USA; 2 Department of Human Genetics, University of Chicago, Chicago, IL 60637 USA; 3 Department of Biostatistics, University of Michigan, Ann Arbor, MI 48109 USA; 4 Center for Statistical Genetics, University of Michigan, Ann Arbor, MI 48109 USA

## Abstract

Single cell RNA sequencing (scRNAseq) technique is becoming increasingly popular for unbiased and high-resolutional transcriptome analysis of heterogeneous cell populations. Despite its many advantages, scRNAseq, like any other genomic sequencing technique, is susceptible to the influence of confounding effects. Controlling for confounding effects in scRNAseq data is a crucial step for accurate downstream analysis. Here, we present a novel statistical method, which we refer to as scPLS (single cell partial least squares), for robust and accurate inference of confounding effects. scPLS takes advantage of the fact that genes in a scRNAseq study often can be naturally classified into two sets: a control set of genes that are free of effects of the predictor variables and a target set of genes that are of primary interest. By modeling the two sets of genes jointly using the partial least squares regression, scPLS is capable of making full use of the data to improve the inference of confounding effects. With extensive simulations and comparisons with other methods, we demonstrate the effectiveness of scPLS. Finally, we apply scPLS to analyze two scRNAseq data sets to illustrate its benefits in removing technical confounding effects as well as for removing cell cycle effects.

## Introduction

Single-cell RNA sequencing (scRNAseq) has emerged as a powerful tool in genomics. While the traditional RNA sequencing, known as the bulk RNAseq, measures gene expression levels averaged across many different cells in a sample of potentially heterogeneous cell population, scRNAseq can measure gene expression levels directly at the single cell resolution. As a result, scRNAseq is less influenced by the variation of cell type and cell composition across different samples–a major confounding in the analyses of bulk RNAseq studies. Because of this benefit and its high resolution, scRNAseq provides unprecedented insights into many basic biological questions that are previously difficult to address. For example, scRNAseq has been applied to classify novel cell subtypes^1,2^ and cellular states^3,4^, reconstruct cell lineage and quantify progressive gene expression during development^5–8^, perform spatial mapping and re-localization^9,10^, identify differentially expressed genes and gene expression modulars^11–13^, and investigate the genetic basis of gene expression variation by detecting heterogenic allelic specific expressions^14,15^.

Like any other genomic sequencing experiment, scRNAseq studies are influenced by many factors that can introduce unwanted variation in the sequencing data and confound the down-stream analysis^16^. However, such unwanted variation are often exacerbated in scRNAseq experiments due to a range of scRNAseq specific conditions that include amplification bias, low amount of input material and low transcript capture efficiency^17^; dropout events that are driven by both biological and technical factors^18,19^; global changes in expression due to transcriptional bursts^20^; as well as changes in cell cycle and cell size^21^. Indeed, adjusting for confounding factors in scRNAseq data has been shown to be crucial for accurate estimation of gene expression levels and successful down-stream analysis^16–18,22,23^. However, depending on the source, adjusting for confounding factors in scRNAseq can be non-trivial. Some confounding effects, such as read sampling noise and drop-out events, are direct consequences of low sequencing-depth, which are random in nature and can be readily addressed by probabilistic modeling using existing statistical methods^18,22–25^. Other confounding effects are inherent to a particular experimental protocol and can cause amplification bias, but can be easily mitigated by using new protocols^26^. Yet other confounding effects are due to observable batches and can be adjusted for by including batch labels and technician ids as covariates or dealt with other statistical methods^27,28^. However, many confounding factors are hidden and are difficult or even impossible to measure. Common hidden confounding factors include various technical artifacts during library preparation and sequencing, and unwanted biological confounders such as cell cycle status. These hidden confounding factors can cause systematic bias, are notoriously difficult to control for, and are the focus of the present study.

To effectively infer and control for hidden confounding factors in scRNAseq studies, we develop a novel statistical method, which we refer to as scPLS (single cell partial least squares). scPLS takes advantage of the fact that genes in a scRNAseq study can often be naturally classified into two sets: a control set of genes that are free of effects of the predictor variables and a target set of genes that are of primary interest. By modeling the two sets of genes jointly using the partial least squares regression, scPLS is capable of making full use of the data to improve the inference of confounding factors. scPLS is closely related to and bridges between two existing subcategories of methods for transcriptome analysis: a subcategory of methods that treat control and target genes in the same fashion (e.g. PCA^29–32^ and LMM^33–35^), and another subcategory of methods that use control genes alone for inferring confounding factors (e.g. RUV^29,36^ and scLVM^37^). By bridging between the two subcategories of methods, scPLS enjoys robust performance across a range of application scenarios. scPLS is also computationally efficient: with a new block-wise expectation maximization (EM) algorithm, it is scalable to thousands of cells and tens of thousands of genes. Using simulations and two real data applications, we show how scPLS can be used to remove confounding effects and enable accurate down-stream analysis in scRNAseq studies. Our method is implemented as a part of the Citrus project and is freely available at: http://chenmengjie.github.io/Citrus/.

The paper is organized as follows. In the Review of Previous Methods section, we provide a brief review of existing statistical methods for removing confounding effects in transcriptome analysis and describe how scPLS is related to and motivated from these methods. In the Method Overview Section, we provide a methodological description of the scPLS model, with inference details provided in the Methods Section. In the Simulations section we present comparisons between scPLS and several existing methods using simulations. In Real Data Applications section, we apply scPLS to two real scRNAseq data sets to remove technical confounding effects or cell cycle effects. Finally, we conclude the paper with a summary and discussion.

## Review of Previous Methods

Many statistical methods have been developed in sequencing- and array-based genomic studies to infer hidden confounding factors and control for hidden confounding effects. Based on their targeted application, these statistical methods can be generally classified into two categories.

The first category of methods are supervised and application-specific: these methods are designed to infer the confounding factors in the presence of a *known* predictor variable, and to correct for the confounding effects without removing the effects of the predictor variable. For example, scientists are often interested in identifying genes that are differentially expressed between two pre-determined treatment conditions or that are associated with a measured predictor variable of interest. To remove the confounding effects in these applications, methods, include SVA^30^, sparse regression models^38,39^, and, more recently, RUV^40,41^, are developed. Although these application-specific methods are widely applied in many genomics studies, their usage is naturally restricted to cases where the primary variable of interest is known. The application-specific methods become inconvenient in cases where there are multiple variables of interest (e.g. in eQTL mapping problems). They also become inapplicable when the primary variable of interest is not observed (e.g. in clustering problems).

The second category of methods are unsupervised, and are designed to infer the confounding factors without knowing or using the predictor variable of interest. Our scPLS belongs to this category. Notable applications of unsupervised methods in scRNAseq studies include cell type clustering and classification^1–8^. Existing unsupervised statistical methods can be further classified into two subcategories. The first subcategory of methods treat all genes in the same fashion and use all of them to infer the confounding factors. For example, the principal component analysis (PCA) or the factor model extracts the principal components or factors from all genes (or all highly variable genes) as surrogates for the confounding factors^29–32^. The inferred factors are treated as covariates whose effects are further removed from gene expression levels before downstream analyses. Similarly, the linear mixed models (LMMs) construct a sample relatedness matrix based on all genes to capture the influence of the confounding factors^33–35^. The relatedness matrix are then included in the downstream analyses to control for the confounding effects. In contrast, the second subcategory of unsupervised methods are recently developed to take advantage of a set of control genes for inferring the confounding factors^29,37^. These methods divide genes into two sets: a control set of genes that are known to be free of effects of interest *a priori* and a target set of genes that are of primary interest. Unlike the first subcategory, the second subcategory of methods treat the two gene sets differently in inferring the confounding factors: the confounding factors are only inferred from the control set, and are then used to remove the confounding effects in the target genes for subsequent downstream analysis. For example, scRNAseq studies often add ERCC spike-in controls prior to the PCR amplification and sequencing steps. The spike-in controls can be used to capture the hidden confounding technical factors associated with the experimental procedures, which are further used to remove technical confounding effects (e.g. reverse transcription or PCR amplification confounding effects) from the target genes^33^. Similarly, most scRNAseq studies include a set of control genes that are known to have varying expression levels across cell cycles. These cell cycle genes can be used to capture the unmeasured cell cycle status of each cell, which are further used to remove cell cycle effects in the target genes^37^. Prominent methods in the second subcategory include the unsupervised version of RUV^29,36^ and scLVM^37^.

The two subcategories of unsupervised methods use different strategies to infer the confounding factors. Therefore, these two sets of methods are expected to perform well in different settings. Specifically, the first subcategory of methods have the advantage of using information contained in all genes to accurately infer the confounding effects. However, when the predictor variable of interest influences a large number of genes, then this subcategory of methods may incorrectly remove the primary effects of interest. On the other hand, the second subcategory of methods infer confounding factors only from the control genes and are thus not prone to mistakenly removing the primary effects of interest. However, these methods overlook one important fact–that the hidden confounding factors not only influence the control genes but also the target genes, i.e. the exact reason that we need to remove such confounding effects in the first place. Because the confounding factors influence both control and target genes, using control genes alone to infer the confounding factors can be suboptimal as it fails to use the information from target genes.

To more effectively infer and control for hidden confounding factors in scRNAseq studies, we develop a novel statistical method, which we refer to as scPLS (single cell partial least squares). scPLS bridges between the two subcategories of unsupervised methods and effectively includes each as a special case. Like the first subcategory of methods, scPLS models both control and target genes jointly to infer the confounding factors. Like the second subcategory of methods, scPLS is capable of taking advantage of a control set to guild the inference of confounding factors. scPLS builds upon the partial least squares regression model and relies on a key modeling assumption that only target genes contain the primary effects of interest or other systematic biological variations. By incorporating such systematic variations in the target genes only, we can jointly model both control and target genes to infer the confounding effects while avoiding mis-removing the primary effects of interest. Therefore, scPLS has the potential to make full use of the data to improve the inference of confounding factors and the removal of confounding effects.

## Results

### scPLS Method Overview

We provide modeling details for scPLS here. While the formulation of scPLS is general, we focus on its application in scRNAseq. The scRNAseq data resembles that of the bulk RNAseq data and consists of a gene expression matrix on *n* cells and $p+q$ genes. We consider dividing the genes into two sets: a control set that contains *q* control genes and a target set that contains *p* genes of primary interest. The control genes are selected based on the purpose of the analysis. For example, the control set would contain ERCC spike-ins if we want to remove technical confounding factors, and would contain cell cycle genes if we want to remove cell cycle effects. We use the following partial least squares regression to jointly model both control and target genes:

1 $${{\bf{x}}}_{{\bf{i}}}={{\boldsymbol{\Lambda }}}_{x}{{\bf{z}}}_{{\bf{i}}}+{{\boldsymbol{\varepsilon }}}_{xi},{{\boldsymbol{\varepsilon }}}_{xi} \sim \,{\rm{MVN}}\,\mathrm{(0},\,{{\boldsymbol{\Psi }}}_{xi})$$

2 $${{\bf{y}}}_{{\bf{i}}}={{\boldsymbol{\Lambda }}}_{y}{{\bf{z}}}_{{\bf{i}}}+{{\boldsymbol{\Lambda }}}_{u}{{\bf{u}}}_{{\bf{i}}}+{{\boldsymbol{\varepsilon }}}_{yi},{{\boldsymbol{\varepsilon }}}_{yi} \sim {\rm{MVN}}\,\mathrm{(0},\,{{\boldsymbol{\Psi }}}_{yi})$$

where for $i$'th individual cell, ${{\bf{x}}}_{{\bf{i}}}$ is a *q*-vector of expression levels for *q* control genes; ${{\bf{y}}}_{{\bf{i}}}$ is a *p*-vector of expression levels for *p* target genes; ${{\bf{z}}}_{{\bf{i}}}$ is ${k}_{z}$-vector of unknown confounding factors that affect both control and target genes; the coefficients of the confounding factors are represented by the $q$ by ${k}_{z}$ loading matrix ${{\boldsymbol{\Lambda }}}_{x}$ for the control genes and the $p$ by ${k}_{z}$ loading matrix ${{\boldsymbol{\Lambda }}}_{y}$ for the target genes; ${{\bf{u}}}_{{\bf{i}}}$ is a ${k}_{u}$-vector of unknown factors in the target genes and potentially represents the predictors of interest or other structured variations (see below); ${{\boldsymbol{\Lambda }}}_{u}$ is a $p$ by ${k}_{u}$ loading matrix; ${{\boldsymbol{\varepsilon }}}_{xi}$ is a $q$-vector of idiosyncratic error with covariance ${{\boldsymbol{\Psi }}}_{xi}=diag({\sigma }_{x1}^{2},\cdots ,{\sigma }_{xq}^{2})$; ${{\boldsymbol{\varepsilon }}}_{yi}$ is a $p$-vector of idiosyncratic error with covariance ${{\boldsymbol{\Psi }}}_{yi}=diag({\sigma }_{y1}^{2},\cdots ,{\sigma }_{yp}^{2})$; MVN denotes the multivariate normal distribution. We assume that ${{\boldsymbol{\varepsilon }}}_{xi}$, ${{\boldsymbol{\varepsilon }}}_{yi}$, ${{\bf{z}}}_{{\bf{i}}}$, and ${{\bf{u}}}_{{\bf{i}}}$ are all independent from each other. Following standard latent factor models, we further assume that ${{\bf{z}}}_{{\bf{i}}} \sim {\rm{MVN}}\,\mathrm{(0},\,{\bf{I}})$ and ${{\bf{u}}}_{{\bf{i}}} \sim {\rm{MVN}}\mathrm{(0,}\,{\bf{I}})$. We model transformed data instead of the raw read counts. We also assume that the expression levels of each gene have been centered to have mean zero, which allows us to ignore the intercept.

**Figure 1:**
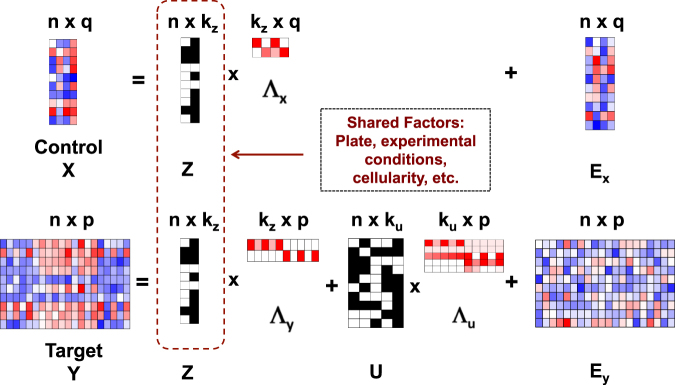
Illustration of scPLS. We model the expression level of genes in the control set $({\bf{X}})$ and genes in the target set $({\bf{Y}})$ jointly. Both control and target genes are affected by the common confounding factors (**Z**) with effects ${{\boldsymbol{\Lambda }}}_{x}$ and ${{\boldsymbol{\Lambda }}}_{y}$ in the two gene sets, respectively. The target genes are also influenced by biological factors (**U**) with effects ${{\boldsymbol{\Lambda }}}_{u}$. The biological factors represent intermediate factors that coordinately regulate a set of genes, and are introduced to better capture the complex variance structure in the target genes. ${{\bf{E}}}_{{\bf{x}}}$ and ${{\bf{E}}}_{{\bf{y}}}$ represent residual errors. scPLS aims to remove the confounding effects ${\bf{Z}}{{\boldsymbol{\Lambda }}}_{y}$ in the target genes.

scPLS includes two types of unknown latent factors. The first set of factors, ${{\bf{z}}}_{{\bf{i}}}$, represents the unknown confounding factors that affect both control and target genes. The effects of ${{\bf{z}}}_{{\bf{i}}}$ on the control and target genes are captured in the loading matrices ${{\boldsymbol{\Lambda }}}_{x}$ and ${{\boldsymbol{\Lambda }}}_{y}$, respectively. We call ${{\bf{z}}}_{{\bf{i}}}$ the confounding factors throughout the text, and we aim to remove the confounding effects ${{\boldsymbol{\Lambda }}}_{y}{{\bf{z}}}_{{\bf{i}}}$ from the target genes. The second set of factors, ${{\bf{u}}}_{{\bf{i}}}$, aims to capture a low dimensional structure of the expression level of $p$ target genes. The factors ${{\bf{u}}}_{{\bf{i}}}$ can represent the unknown predictor variables of interest, specific experimental perturbations, cell subpopulations, gene signatures or other intermediate factors that coordinately regulate a set of genes. Therefore, the factors ${{\bf{u}}}_{{\bf{i}}}$ can be interpreted as cell subtypes, treatment status, transcription factors or regulators of biological pathways in different studies^42–46^. Although ${{\bf{u}}}_{{\bf{i}}}$ could be of direct biological interest in many data sets, we do not explicitly examine the inferred ${{\bf{u}}}_{{\bf{i}}}$ here. Rather, we view modeling ${{\bf{u}}}_{{\bf{i}}}$ in the target genes as a way to better capture the complex variance structure there and to facilitate the precise estimation of confounding factors ${{\bf{z}}}_{{\bf{i}}}$. For simplicity, we call ${{\bf{u}}}_{{\bf{i}}}$ the biological factors throughout the text, though we note that ${{\bf{u}}}_{{\bf{i}}}$ could well represent non-biological processes such as treatment or environmental effects. Thus, the expression levels of the control genes can be described by a linear combination of the confounding factors ${{\bf{z}}}_{{\bf{i}}}$ and residual errors; the expression levels of the target genes can be described by a linear combination of the confounding factors ${{\bf{z}}}_{{\bf{i}}}$, the biological factors ${{\bf{u}}}_{{\bf{i}}}$ and residual errors. For both types of confounding factors, we are interested in inferring the factor effects ${{\boldsymbol{\Lambda }}}_{y}{{\bf{z}}}_{{\bf{i}}}$ and ${{\boldsymbol{\Lambda }}}_{u}{{\bf{u}}}_{{\bf{i}}}$ rather than the individual factors ${{\bf{z}}}_{{\bf{i}}}$ and ${{\bf{u}}}_{{\bf{i}}}$. Therefore, unlike in standard factor models, we are not concerned with the identifiability of the factors. Figure 1 shows an illustration of scPLS.

scPLS is closely related to the two subcategories of unsupervised methods described in the previous Section. Specifically, without the biological effects term ${{\boldsymbol{\Lambda }}}_{u}{{\bf{u}}}_{{\bf{i}}}$, scPLS effectively reduces to the first subcategory of methods that treat all genes in the same fashion for inferring the confounding factors. Without the Equation 2 term, scPLS effectively reduces to the second subcategory of methods that use only control genes for inference. (Note that, after inferring the confounding factors ${{\bf{z}}}_{i}$ from Equation 1, the second subcategory of methods still use a reduced version of Equation 2 without the biological effects term ${{\boldsymbol{\Lambda }}}_{u}{{\bf{u}}}_{{\bf{i}}}$ to remove the confounding effects.) By including both modeling terms, scPLS can robustly control for confounding effects across a range of scenarios. Therefore, scPLS provides a flexible modeling framework that effectively includes the two subcategories of unsupervised methods as special cases and has the potential to outperform these previous methods.

### Simulations

We performed a simulation study to compare scPLS with other methods. Specifically, we simulated gene expression levels for 50 control genes and 1,000 target genes for 200 cells. These 200 cells come from two equal-sized groups, representing two treatment conditions or two cell subpopulations. Among the 1,000 target genes, only 200 of them are differentially expressed (DE) between the two groups and thus represent the signature of the two groups. The effect sizes of the DE genes were simulated from a normal distribution and we scaled the effects further so that the group label explains twenty percent of phenotypic variation (PVE) in expression levels in the DE genes. In addition to the group effects, we set ${k}_{z}=\mathrm{2,}\,{k}_{u}=5$ and simulated each element of ${{\bf{z}}}_{{\bf{i}}}$ and ${{\bf{u}}}_{{\bf{i}}}$ from a standard normal distribution. We simulated each element of ${{\rm{\Lambda }}}_{x}$ from $N(-\mathrm{0.25,}\,{\sigma }_{l}^{2})$ and each element of ${{\boldsymbol{\Lambda }}}_{y}$ from $N\mathrm{(0.25,}\,{\sigma }_{l}^{2})$. Note that ${{\boldsymbol{\Lambda }}}_{x}$ and ${{\boldsymbol{\Lambda }}}_{y}$ were simulated differently to capture the fact that the effect sizes of the confounding factors could be different for control and target genes. We simulated each element of ${{\boldsymbol{\Lambda }}}_{u}$ from $N\mathrm{(0,}\,{\sigma }_{b}^{2})$. We simulated each element of ${\varepsilon }_{xi}$ and ${\varepsilon }_{yi}$ from a standard normal distribution. We set ${\sigma }_{l}^{2}=0.4$ and ${\sigma }_{b}^{2}=0.6$ to ensure that, in non-DE genes, the confounding factors ${{\bf{z}}}_{{\bf{i}}}$ explain 20% PVE in either the control or the target genes; the biological factors ${{\bf{u}}}_{{\bf{i}}}$ explain 30% PVE of the target genes; and the residual errors to explain the rest of PVE. To vary signal strength in the data, we also created a series of sub data sets by varying the number of non DE genes in the data, so that the proportion of variance explained by DE genes in total equal to a fixed percentage (PDE, in the range of 20–100%, with 10% increments). After we simulated gene expression levels, we further converted these continuous values into count data by using a Poisson distribution: the final observation for *i*th cell and *j*th gene ${c}_{ij}$ is from ${c}_{ij} \sim {\rm{Poi}}(N\,\exp (\mu +{w}_{ij}))$, with ${w}_{ij}$ being the continuous gene expression levels simulated above and $N=\mathrm{500000,}\,\mu =\,\mathrm{log}\,\mathrm{(10/500000)}$, which ensures an average read count of 10. Note that, because of the residual errors, the resulting count data are over-dispersed with respect to a Poisson distribution.

We considered three different simulation scenarios. In scenario I, the confounding factors ${{\bf{z}}}_{{\bf{i}}}$ are independent of group labels. In scenario II, the confounding factors are correlated with group labels. To simulate correlated data, we simulated each element of ${{\bf{z}}}_{{\bf{i}}}$ from $N\mathrm{(0,}\,\mathrm{1)}$ if the corresponding sample belongs to the first group, but from $N(-0.25,1)$ if the corresponding sample belongs to the second group. Finally, we also considered a scenario III where there is no biological factor (i.e. data were simulated effectively under the PCA modeling assumption and all genes could be used to infer the confounding factors). We performed 10 simulation replicates for each scenario. For scenario I and II, we further introduced dropout events that are commonly observed in scRNAseq data. This was done by going through one gene at a time and setting the expression level for $j$ th gene ${c}_{ij}$ to zero with probability ${\pi }_{ij}$ that depends on the expression level through $log\frac{{\pi }_{ij}}{1-{\pi }_{ij}}={c}_{ij}$.

We compared scPLS to four different methods: (1) PCA and (2) LMM (implemented in GEMMA^47,48^) use all genes to infer the confounding effects; while (3) RUVseq (version 1.2.0); which we simply refer to as RUV in the following text) and (4) scLVM (version 0.99.1) use only control genes to infer the confounding effects. We note that while some of these methods are developed not specifically for scRNAseq, these methods represent a range of strategies to deal with confounding factors. We used default settings in all the above methods. We used the count data directly for RUV and used log transformed data (i.e. $\mathrm{log}({c}_{ij}+\mathrm{1)}$) for all other methods. For PCA and RUV, we set the number of latent factors to be the true number (i.e. 2). Such number is determined automatically by the software itself for scLVM, and is not needed for LMM. We compared different methods based on clustering performance. In particular, for each of these methods, we obtained corrected data and applied k-means method to cluster cells into two subpopulations. We the compared the clusters inferred from the corrected data with the truth and used adjusted rand index (ARI) to measure clustering performance. ARI is computed across a range of signal strength that is measured as PDE explained above. Intuitively, if a method performs well in removing confounding factors, then the corrected data from this method can be used to better infer the two cell subpopulations and thus yields a higher ARI score.

**Figure 2:**
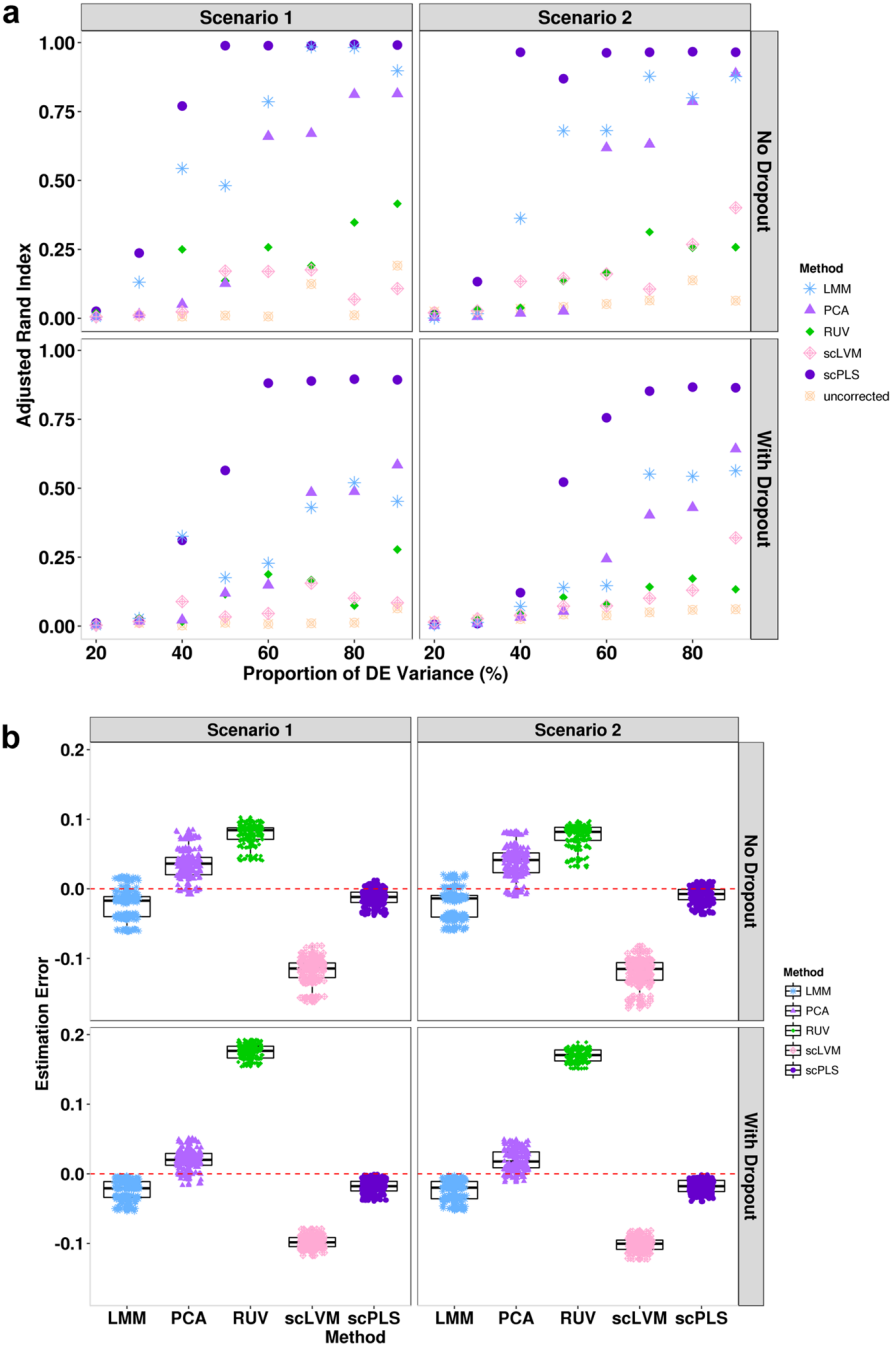
Method comparison in simulations. Clustering analysis using scPLS-corrected data achieves higher Adjusted Rand Index (ARI) than using LMM-, PCA-, RUV- and scLVM-corrected data or uncorrected data in both scenario I with (**a**) or without drop-out (**c**) and scenario II with (**b**) or without drop-out (**d**) across a range of signal strength. ARI is averaged across ten simulation replicates. x-axis shows the signal strength, which are measured as the percentage of DE genes variance out of all genes. (**c**) Sensitivity analysis shows that, scPLS maintains a high ARI (y-axis) when a smaller subset of control genes are used (*q* = 10, 20, 30 or 40 instead of 50).

Overall, scPLS performs the best in both scenarios I and II, with or without dropout events (Fig. 2a). The addition of dropout events in either of the two scenarios reduces the performance of all methods but does not change their relative rank of performance. The superior performance of scPLS also suggests that properly using both control and target genes can lead to effective removal of confounding effects. Among the rest of the methods, PCA and LMM performs better than RUV and scLVM, suggesting that target genes contain a substantial amount of information for removing confounding effects. Beside the comparison of clustering performance, for each gene in turn, we also used different methods to estimate the proportion of gene expression variance contributed by confounding factors. Consistent with the clustering performance comparison, we found that scPLS also yielded more accurate proportion of variance estimates (Fig. 2b).

**Figure 3:**
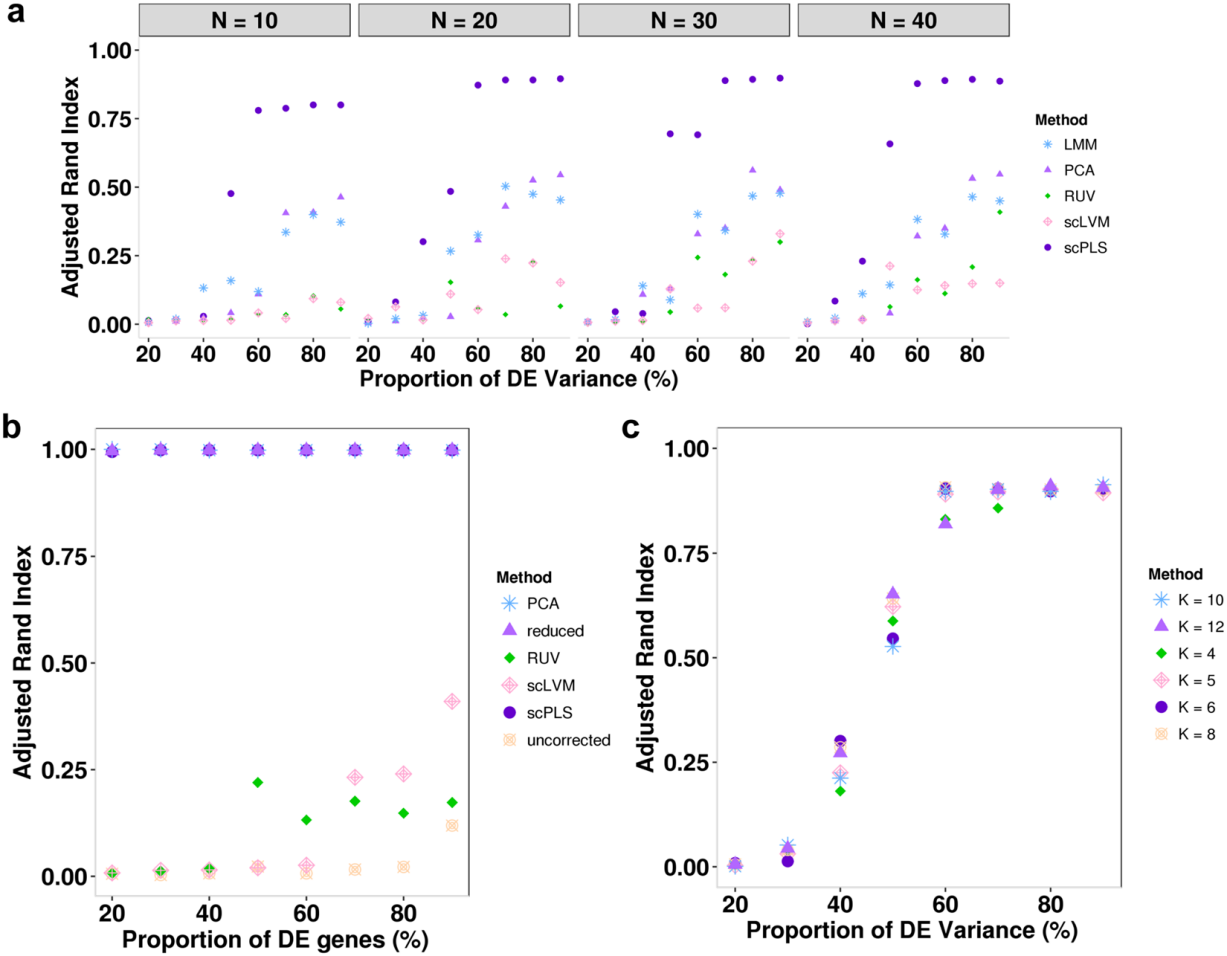
Method comparison in simulations (continued). (**a**) Error in estimating the proportion of variance contributed by confounding factors across genes using data corrected by different methods. Error is computed as the difference between the estimated proportion and the true proportion. (**b**) scPLS performs well in Scenario III when the model is misspecified (with true ${k}_{u}=0$). (**c**) scPLS is robust with respect to ${k}_{u}$, as the ARI remains similar when a different number of biological factors is used (*k*_*u*_ = 2, 4, 6, 8) in Scenario I with dropout. x-axis shows the signal strength, which are measured as the percentage of DE genes variance out of all genes.

To examine the robustness of scPLS, we applied scPLS to the same data but with a reduced number of control genes (Fig. 3a). Because scPLS does not completely rely on the information contained in the control genes, it achieves robust performance even if we only use a much smaller subset of control genes. We also examined the performance of scPLS in Scenario III where there is no biological effects (Fig. 3b) and found that scPLS performs well there. As it is often unknown whether a low-rank structural variation exists in a real data set, our simulation suggests that we can always include the biological factors ${{\bf{u}}}_{{\bf{i}}}$ in the model even in the absence of such factors. In addition, scPLS is not sensitive with respect to the number of biological factors used in fitting the model, and achieves similar power for a range of reasonable ${k}_{u}$ values (Fig. 3c).

### Real Data Applications

Next, we applied scPLS to two real data sets. The first dataset is used to demonstrate the effectiveness of scPLS in removing the technical confounding effects by using ERCC spike-ins. Removing technical confounding effects is a common and important task in transcriptome analysis. The second dataset is used to demonstrate the effectiveness of scPLS in removing cell cycle effects by using a known set of cell cycle genes. Removing cell cycle effects can reveal gene expression heterogeneity that is otherwise obscured.

### Removing Technical Confounding Factors

The first dataset consists of 251 samples from^22^. Among these, 119 are mouse embryonic stem cells (mESCs), including 74 mESCs cultured in a two-inhibitor (2i) medium and 45 mESCs cultured in a serum medium. The remaining 132 cells are control “cells” that are obtained by mixing single cells cultured in each condition (i.e. these control “cells” are similar to bulk seq data in terms of consisting a mixture of cell types, but are prepared and sequenced using single cell protocol). The control cells include 76 cells cultured in 2i and 56 cells cultured in serum. Because the control cells are homogeneous within each culture condition, when we cluster these cell, we would expect the only true cluster detectable among these cells is the culture condition. Therefore, we decide to focus on these control cells to compare the performance of different methods for removing technical effects.

We obtained the raw UMI counts data directly from the authors. The data contains measurements for 92 ERCC spike-ins and 23,459 genes. Due to the low coverage of this dataset (median coverage equals one), we filtered out lowly expressed genes and selected only genes that have at least five counts and spike-ins that have at least one count in more than a third of the cells. This filtering step resulted in a total of 32 ERCC spike-ins that were used as the controls and 2,795 genes that were used as the targets.

As in the simulations, we log transformed the count data and centered the transformed values for scPLS, PCA, LMM and scLVM. We used the count data for RUV. In this data, scPLS infers ${k}_{z}=1$ confounding factors and ${k}_{u}=1$ biological factors. In the target genes, the confounding factors and structured biological factors explain a median of 18% and 30% of gene expression variance, respectively. The PVE by the confounding and biological factors can be as high as 73.7% and 77.9%, respectively, in the target genes.

We applied scPLS and the other four methods to remove confounding effects in the data. Since control cells are homogeneous within each culture condition, we reasoned that if the method is effective in removing confounding effects, then the corrected data from the corresponding method could be used to better reveal two clusters that correspond to the two known culture conditions. For the clustering analysis, we applied the four different clustering approaches on the uncorrected or corrected data from different methods. The four clustering approaches include: (1) kmeans, where we applied the k-means method directly on the uncorrected or corrected data; (2) PCA, where we extracted the top five PCs from either the uncorrected or corrected data and then applied the k-means method using the top PCs; (3) tSNE, where we used tSNE to either the uncorrected or corrected data and then applied the k-means method on the extracted tSNE factors; (4) SC3, where we used a recently developed state-of-the-art single cell clustering method single cell census clustering (SC3)^49^. For all these clustering approaches, we set the number of clusters to two and measured clustering performance by the adjusted Rand Index (ARI). The results are shown in Table 1 and are overall consistent with the simulations. Specifically, scPLS outperforms the other methods in three out of the four clustering approaches. scPLS performs slightly worse than RUV when tSNE was used to cluster data–but tSNE works extremely poorly in this data presumably because tSNE’s non-linearity assumption does not fit the data well.

**Table 1:** Clustering performance on the uncorrected data or data corrected by different methods (columns). Different clustering approaches (rows) are applied in order to examine the robustness of the comparison results. Clustering performance is measured by the adjusted Rand Index. All performance measurements are averaged across 10 runs and are multiplied by a factor of 100. The top performer is colored blue.

	uncorrected	scPLS	RUV	LMM	PCA	scLVM
kmeans	59	100	31	67	42	4
PCA	59	100	31	67	67	0
tSNE	42	44	46	44	36	0
SC3	100	100	97	91	80	2

### Removing Cell Cycle Effects

**Figure 4:**
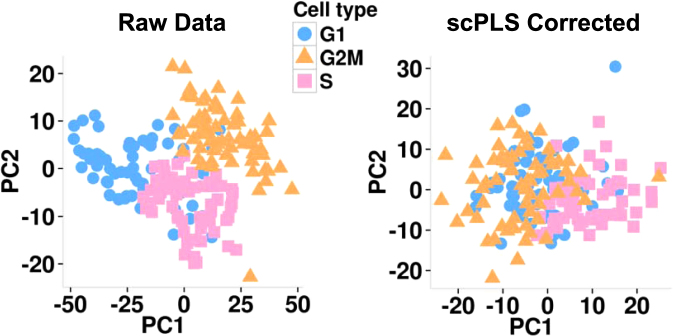
PCA plots for the uncorrected data and scPLS corrected data in the second dataset. In the uncorrected data, there is a clear separation of cells by cell-cycle stage. Such separation of cells is no longer observed in the scPLS corrected data.

Our method can also be used to remove cell cycle effects. To demonstrate its effectiveness there, we applied scPLS and several other methods to a second dataset that was used for demonstrating cell cycle influence^37^. This dataset contains gene expression measurements on 9,570 genes from 182 embryonic stem cells (ESCs) with pre-determined cell-cycle phases (G1, S and G2M). The uncorrected data we obtained are already pre-processed by the original study to remove the technical effects and are thus continuous. Therefore, we did not apply RUV here. To remove cell cycle effects, we used 629 annotated cell-cycle genes as controls and the other genes as targets. scPLS infers ${k}_{z}=1$ cell cycle confounding factors, and ${k}_{u}=1$ biological factors. These factors explain a median of 0.4% and 0.1% of gene expression variance, respectively. The PVE by cell cycle factors and biological factors can be as high as 7% and 2%, respectively. We visualized the uncorrected data and scPLS corrected data on a PCA plot (Fig. 4). In the uncorrected data, there is a clear separation of cells according to cell-cycle stage. Such separation of cells is not observed in the corrected data, indicating that the cell cycle related expression signature is effectively removed.

We compared scPLS and the other three methods in their effectiveness in removing cell cycle effects. Following the original study^37^, we evaluated method performance based on the following criteria. Specifically, we computed for each gene the proportion of expression variance explained by the cell cycle factor. We denote this quantity as PVEi, which stands for inferred PVE. Because the cell-cycle stage of each cell had been experimentally determined in this data set, we further computed the variance explained by the true cell cycle labels. We denote this quantity as PVEt, which stands for true PVE. For scPLS, PVEi and PVEt are highly correlated (${r}^{2}=0.94$), demonstrating the efficacy of scPLS. The correlation remains the same whether we use the full control set or with a subset of 300 controls. The correlation between PVEi and PVEt in scPLS is slightly higher, with statistical significance, than scLVM (${r}^{2}=0.92$; p-value $< \,{10}^{-16}$ comparing scPLS vs scLVM), LMM (${r}^{2}=0.92$; p-value $< \,{10}^{-16}$ comparing scPLS vs LMM), and PCA (${r}^{2}=0.92$; p-value $< \,{10}^{-16}$ comparing scPLS vs PCA). In addition, as an alternative measurement, the median of the absolute difference between PVEi and PVEt across genes from scPLS, scLVM, LMM and PCA are 0.018, 0.023, 0.019 and 0.019, respectively, again supporting a small advantage of scPLS. However, we do want to acknowledge that all methods work reasonably well in this data (which is consistent with the low variance explained by the confounding factors), suggesting that removing cell cycle effects is a relatively trivial task at least in this data set.

## Discussion

We have presented scPLS for removing hidden confounding effects in scRNAseq studies. scPLS models both control and target genes jointly to infer the confounding factors and shows robust performance across a range of application scenarios. With simulations and applications to two real data sets, we have demonstrated its effectiveness for removing technical confounding effects or cell cycle effects in scRNAseq studies.

Although we have focused on its applications to scRNAseq studies, scPLS can be readily applied to other genomic sequencing studies. For instance, our method can be used to remove confounding effects from gene expression levels in bulk RNAseq studies^50^ or from methylation levels in bisulfite sequencing studies^51^. The main requirement of our method is a set of pre-specified control genes that are measured together with the target genes in the sequencing studies. It is often straightforward to obtain such control genes. For example, many scRNAseq studies include a set of ERCC spike-in controls that could be used to model and remove technical confounding effects^33^. Even when such ERCC spike-in controls are not present or when they are unreliable^29^, we can select a known set of house-keeping genes as controls to remove technical confounding^29^. Similarly, we can use a set of known cell cycle genes to remove cell cycle effects. Importantly, the performance of scPLS is robust to the number of genes included in the control set and yields comparable results even when a much smaller number of control genes is used. This is because scPLS not only uses information from control genes but also relies on information from target genes. Insensitivity to the control set makes scPLS especially suited to removing confounding factors in studies where a control set is not clearly defined. Because of its effectiveness and robustness, we expect scPLS to be useful in removing confounding effects in a wide variety of sequencing studies.

One important feature of scPLS is that it includes a low-rank component to model the structured biological variation often observed in real data. By decomposing the (residual) gene expression variation into a low-rank structured component that is likely to be contributed by a sparse set of biological factors, and an unstructured component that reflects the remaining variation, scPLS can better model the residual error structure for accurate inference of confounding effects. Although here we have focused on using the biological factors to better infer the confounding effects, we note that the low-rank biology factors themselves could be of direct interest. In fact, low-rank factors inferred from many data sets using standard factor models have been linked to important biological pathways or transcription factors^42–46^. Inferring the biological factors using scPLS is not feasible at the moment, however: because of model identifiability, scPLS can only be used to infer the biological effects (i.e. ${{\boldsymbol{\Lambda }}}_{u}{{\bf{u}}}_{{\bf{i}}}$) but not the biological factors (i.e. ${{\bf{u}}}_{{\bf{i}}}$). That said, additional assumptions can be made on the structure of the factors or the factor loading matrices to make factor inference possible^52^. For example, we could impose sparsity assumptions on the low-rank factors to facilitate the inference of a parsimonious set of biological factors. Exploring the use of biological factors in scPLS is an interesting avenue for future research.

We have been mainly focused on comparing the performance of different confounding effects removing methods by evaluating the clustering performance as the target downstream analysis. It has been well recognized that the choice of data normalization in scRNA-Seq is highly dependent on the specific biological question and the target downstream analysis^53^. Indeed, different downstream analysis (e.g. differential expression, lineage reconstruction, detecting allele-specific expression, spatial reconstruction etc.) can be affected differently by different choices of normalization. While evaluating the performance of various confounding effects removing methods for other downstream analysis is beyond the scope of the present study, we acknowledge that the “best” confounding effects removing method may vary depending on the question of interest. Therefore, it would be important to evaluate the performance of scPLS in other analysis settings in future studies. Nevertheless, we believe scPLS represent an important addition to the existing tools for removing confounding effects. Finally, in simulations we have also mainly focused on using the k-means clustering method to evaluate the clustering performance. Many other clustering methods are being developed recently, some of which are specifically targeted to single cell RNAseq studies. Those methods include RaceID^54^, SCUBA^55^, SNN-Cliq^56^, ZIFA^57^, t-SNE^4^, SC3^49^; just to name a few. Because scPLS does not rely on a particular clustering method, scPLS can be paired with any clustering methods to take advantage of their benefits. Indeed, we have applied different clustering approaches to measure the performance of scPLS and other methods for removing confounding effects in the real data and obtained consistent results.

Like many other methods for scRNAseq^21^ or bulk^58,59^ RNAseq studies, scPLS requires a data transformation step that converts the count data into quantitative expression data. Different transformation methods can affect the interpretation of the data and are advantageous in different situations^16^. Because scPLS does not rely on a particular transformation procedure, scPLS can also be paired with any transformation methods to take advantage of their benefits. One potential disadvantage of scPLS is that it does not model raw count data directly. In bulk RNAseq studies, despite the count nature of sequencing data, it has been show that there is often a limited advantage of modeling the raw read counts directly, at least for RNAseq studies^60,61^. Statistical methods that convert and model the quantitative expression data have been shown to be robust^58,59^ and most large scale bulk RNAseq studies in recent years have used transformed data instead of count data^31,62–64^. However, we note that, unlike bulk RNAseq studies, single cell RNAseq data often come with low read depth. In low read depth cases, modeling count data while accounting for over-dispersion or dropout events in single cell RNAseq studies may have added benefits^17,18^. Therefore, extending our framework to modeling count data^65,66^ is another promising avenue for future research.

## Methods

### EM Algorithms for scPLS

We develop an expectation-maximization (EM) algorithm for inference in scPLS. Specifically, we first initialize the factor loading matrices $({{\boldsymbol{\Lambda }}}_{x},{{\boldsymbol{\Lambda }}}_{y},{{\boldsymbol{\Lambda }}}_{u})$ based on sequential single value decompositions on the gene expression matrices $({\bf{X}}=({{\bf{x}}}_{{\bf{1}}},\cdots ,{{\bf{x}}}_{{\bf{q}}}),{\bf{Y}}=({{\bf{y}}}_{{\bf{1}}},\cdots ,{{\bf{y}}}_{{\bf{p}}}))$ (Algorithm 1). Afterwards, we treat the latent factors $({{\bf{w}}}_{{\bf{i}}}={({{\bf{z}}}_{{\bf{i}}}^{T},{{\bf{u}}}_{{\bf{i}}}^{T})}^{T})$ as missing data, use an iterative procedure to compute the expectation of the factors conditional on each individual cell data $({{\bf{v}}}_{{\bf{i}}}=({{\bf{x}}}_{{\bf{i}}}^{T},{{\bf{y}}}_{{\bf{i}}}^{T}{)}^{T})$ in turn in the E-step, and then update the factor loading matrices $$({\boldsymbol{\Lambda }}={(\begin{array}{cc}{{\boldsymbol{\Lambda }}}_{x} & {\bf{0}}\\ {{\boldsymbol{\Lambda }}}_{y} & {{\boldsymbol{\Lambda }}}_{u}\end{array})}^{T},\,{{\bf{v}}}_{{\bf{i}}}=(\begin{array}{c}{{\bf{z}}}_{{\bf{i}}}\\ {{\bf{u}}}_{{\bf{i}}}\end{array}))$$ by merging information across all individuals in the M-step (Algorithm 2). We list the EM algorithm below, with detailed derivation provided later.

**Figure:**
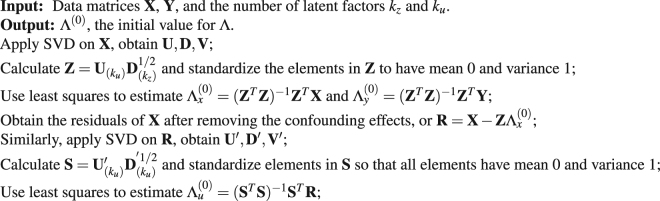
**Algorithm 1:** Initializer of EM algorithms for scPLS.

**Figure:**
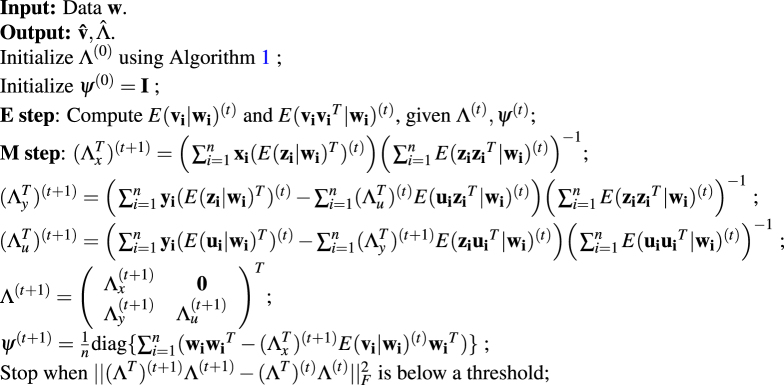
**Algorithm 2:** Naive EM algorithm for scPLS.

**Figure 5:**
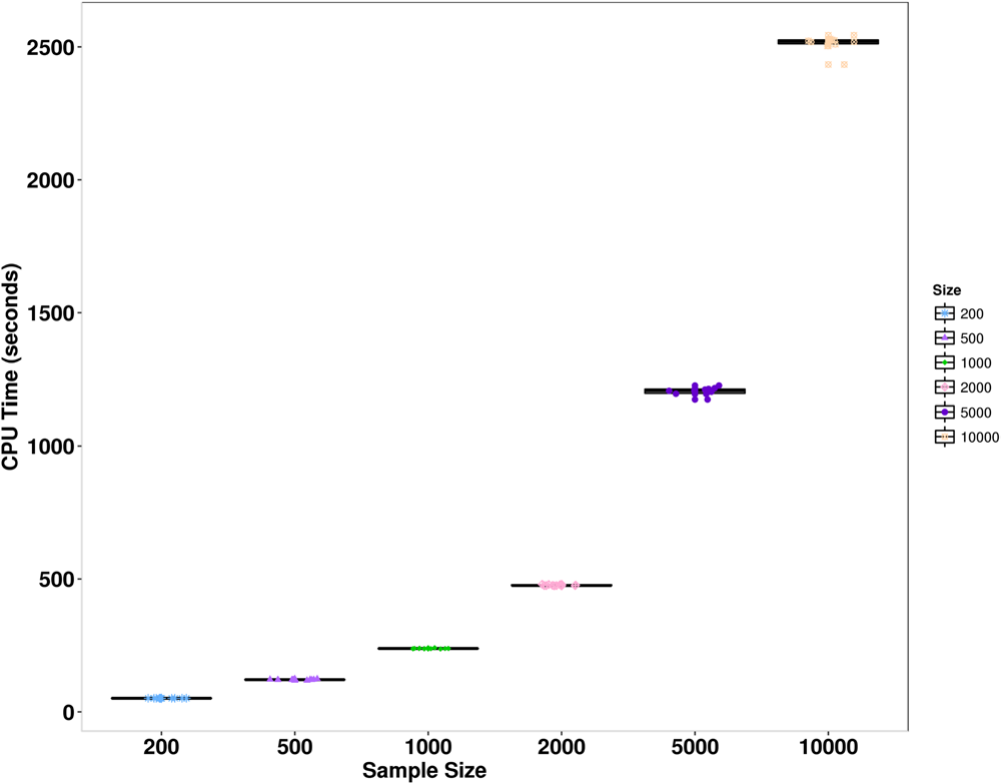
Computing time of the EM-in-chunks algorithm for analyzing single cell data sets of varying sample sizes. Computational time, in seconds (y-axis), were measured on data sets with a fixed number of genes (=1000) but varying number of single cells (x-axis). Ten replicates were performed for each setting on an Intel Xeon E5-2670 2.6 GHz CPU.

We refer to the above algorithm (Algorithm 2) as the naive EM algorithm. The naive EM algorithm is computationally expensive: it scales quadratically with the number of genes and linearly with the number of cells/samples. To improve the computational speed, we develop a new EM-in-chunks algorithm (Algorithm 3). Our algorithm is based on the observation that the expression levels of the target genes are determined by the same set of underlying factors and that these factors can be estimated accurately even with a small subset set of target genes. This allows us to randomly divide target genes into dozens of chunks, compute the expectation of the factors in each chunk separately in the E-step, and then average these expectations across chunks. With the averaged expectations, we then update the factor loading matrices in the M-step. Thus, our new algorithm modifies the E-step in the naive algorithm and becomes $K$ times faster than the naive one, where $K$ is the number of chunks. This same idea has also been applied in the ZIFA algorithm^57^. Simulations (detailed in the simulations Section) show that our EM-in-chunks algorithm yields almost comparable results to the naive EM algorithm with respect to estimation errors, but can be close to an order of magnitude faster (Table 2). With the EM-in-chunks algorithm, our method is easily scalable to handle tens of thousands of cells (Fig. 5). For example, on a single Xeon desktop CPU, we can analyze 10,000 cells and 1,000 genes using our method in approximately 40 min. Therefore, we apply the EM-in-chunks algorithm with chunk size 500 throughout the rest of the paper.

**Figure:**
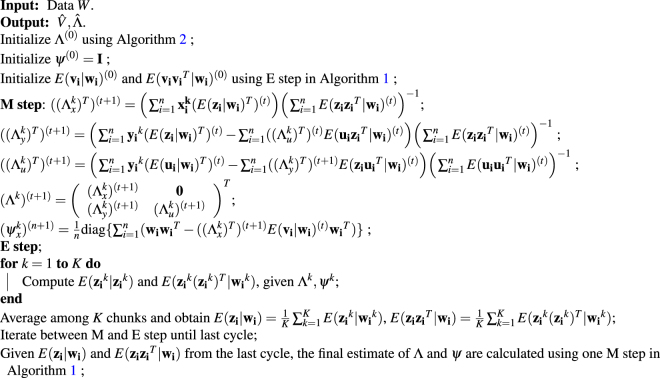
**Algorithm 3:** EM-in-chunks algorithm for scPLS.

**Table 2:** Comparison of the naive EM algorithm and the EM-in-chunks algorithm in terms of accuracy and speed. The EM-in-chunks algorithm uses either a chunk size of 500 genes or a chunk size of 1,000 genes. Accuracy is measured by the estimation error of the loading matrix in terms of the normalized Frobenius norm (i.e. $\sqrt{||{\Lambda }_{x}-{\hat{\Lambda }}_{x}{||}_{F}/n}$). Because of the dimensionality of the loading matrix, the estimation error is not guaranteed to decrease with increasing sample size *n*. Speed is measured by CPU time in seconds for 100 iterations on an Intel Xeon E5-2670 2.6 GHz CPU. Standard deviations across 10 replicates are listed inside parenthesis. *s*: number of genes per chunk. *n*: the number of cells. *p*: the number of genes in the target set. The number of genes in the control set is *q* = 50 in all simulations.

*n*	*p*	Naive EM		EM-in-chunks $(s=\mathrm{1,000})$		EM-in-chunks $(s=500)$	
		Accuracy	CPU time	Accuracy	CPU time	Accuracy	CPU time
200	2000	67.29 (5.33)	244.9 (0.35)	73.32 (6.09)	103.8 (0.06)	75.6 (6.52)	57.78 (0.03)
200	4000	135.07 (10.48)	964.39 (1.95)	144.00 (13.38)	216.25 (0.71)	148.57 (14.11)	123.06 (0.20)
400	2000	72.96 (5.58)	467.6 (0.97)	66.98 (5.15)	203.09 (1.09)	53.48 (4.61)	110.43 (0.10)
400	4000	95.5 (7.41)	1834.86 (3.5)	101.8 (9.46)	422.74 (4.84)	105.05 (9.97)	236.23 (0.48)

Finally, we use the Bayesian information criterion (BIC) to determine the number of confounding factors ${k}_{z}$ and the number of biological factors ${k}_{u}$. Specifically, we evaluate the likelihood on a grid of ${k}_{z}$ (1 to 3) and ${k}_{u}$ values (1 to 10) and choose the optimal combination that minimizes the BIC. After estimating the model parameters on the optimal set of ${k}_{z}$ and ${k}_{u}$, we use the residuals ${\hat{{\bf{y}}}}_{{\bf{i}}}={{\bf{y}}}_{{\bf{i}}}-{\hat{{\boldsymbol{\Lambda }}}}_{{\bf{y}}}{\hat{{\bf{z}}}}_{{\bf{i}}}$ as the de-noised values for subsequent analysis. Note that the residuals are only free of the confounding effects ${{\boldsymbol{\Lambda }}}_{y}{{\bf{z}}}_{{\bf{i}}}$ but still contain the biological effects ${{\boldsymbol{\Lambda }}}_{u}{{\bf{u}}}_{{\bf{i}}}$.

### EM Algorithm Derivation

To derive the EM algorithm, we first integrate out the latent variables ${{\bf{z}}}_{{\bf{i}}}$ and ${{\bf{u}}}_{{\bf{i}}}$ and obtain

3 $$P({{\bf{x}}}_{{\bf{i}}}|{{\boldsymbol{\Lambda }}}_{x},{{\boldsymbol{\psi }}}_{x})=MVN\mathrm{(0,}\,{{\boldsymbol{\psi }}}_{x}+{{\boldsymbol{\Lambda }}}_{x}^{T}{{\boldsymbol{\Lambda }}}_{x}),$$

4 $$P({{\bf{y}}}_{{\bf{i}}}|{{\boldsymbol{\Lambda }}}_{y},{{\boldsymbol{\Lambda }}}_{u},{{\boldsymbol{\psi }}}_{y})=MVN\mathrm{(0,}{{\boldsymbol{\psi }}}_{y}+{{\boldsymbol{\Lambda }}}_{y}^{T}{{\boldsymbol{\Lambda }}}_{y}+{{\boldsymbol{\Lambda }}}_{u}^{T}{{\boldsymbol{\Lambda }}}_{u}\mathrm{).}$$

The latent variable ${{\bf{x}}}_{{\bf{i}}}$ and ${{\bf{z}}}_{{\bf{i}}}$ follow a joint normal distribution

5 $$(\begin{array}{c}{{\bf{x}}}_{{\bf{i}}}\\ {{\bf{z}}}_{{\bf{i}}}\end{array}) \sim MVN((\begin{array}{c}{\bf{0}}\\ {\bf{0}}\end{array}),\begin{array}{cc}{{\boldsymbol{\psi }}}_{x}+{{\boldsymbol{\Lambda }}}_{x}^{T}{{\boldsymbol{\Lambda }}}_{x} & {{\boldsymbol{\Lambda }}}_{x}\\ {{\boldsymbol{\Lambda }}}_{x}^{T} & {\bf{I}}\end{array}){\rm{.}}$$

Denoting $${\boldsymbol{\Lambda }}={(\begin{array}{cc}{{\boldsymbol{\Lambda }}}_{x} & {\bf{0}}\\ {{\boldsymbol{\Lambda }}}_{y} & {{\boldsymbol{\Lambda }}}_{u}\end{array})}^{T},\,{{\bf{v}}}_{{\bf{i}}}=(\begin{array}{c}{{\bf{z}}}_{{\bf{i}}}\\ {{\bf{u}}}_{{\bf{i}}}\end{array})$$, and $${\boldsymbol{\psi }}=(\begin{array}{cc}{{\boldsymbol{\psi }}}_{x} & {\bf{0}}\\ {\bf{0}} & {{\boldsymbol{\psi }}}_{y}\end{array})$$, we can re-write $${{\bf{w}}}_{{\bf{i}}}=(\begin{array}{c}{{\bf{x}}}_{{\bf{i}}}\\ {{\bf{y}}}_{{\bf{i}}}\end{array})$$ as ${{\bf{w}}}_{{\bf{i}}}={{\boldsymbol{\Lambda }}}^{T}{{\bf{v}}}_{{\bf{i}}}+{\boldsymbol{\psi }}$. The variables ${{\bf{w}}}_{{\bf{i}}}$ and ${{\bf{v}}}_{{\bf{i}}}$ then follow a joint normal distribution

6 $$(\begin{array}{c}{{\bf{w}}}_{{\bf{i}}}\\ {{\bf{v}}}_{{\bf{i}}}\end{array}) \sim MVN((\begin{array}{c}{\bf{0}}\\ {\bf{0}}\end{array}),\begin{array}{cc}(\begin{array}{cc}{{\boldsymbol{\psi }}}_{y} & {\bf{0}}\\ {\bf{0}} & {{\boldsymbol{\psi }}}_{x}\end{array})+{{\boldsymbol{\Lambda }}}^{T}{\boldsymbol{\Lambda }} & {\boldsymbol{\Lambda }}\\ {{\boldsymbol{\Lambda }}}^{T} & {\bf{I}}\end{array}){\rm{.}}$$

We view the latent factors ${{\bf{v}}}_{{\bf{i}}}$ as the missing data. In the E step, we calculate the expectation of the log likelihood function for complete data. The expectation is taken with respect to the conditional distribution of ${{\bf{v}}}_{{\bf{i}}}$ given ${{\bf{w}}}_{{\bf{i}}}$

7 $$\begin{array}{c}E(\mathrm{log}\,l({\bf{v}},{\bf{w}})|{\bf{w}})=-\frac{1}{2}\sum _{i\mathrm{=1}}^{n}E[{{{\bf{v}}}_{{\bf{i}}}}^{T}{\boldsymbol{\Lambda }}{{\boldsymbol{\psi }}}^{-1}{{\boldsymbol{\Lambda }}}^{T}{{\bf{v}}}_{{\bf{i}}}-2{{{\bf{v}}}_{{\bf{i}}}}^{T}{\boldsymbol{\Lambda }}{{\boldsymbol{\psi }}}^{-1}{{\bf{w}}}_{{\bf{i}}}|{{\bf{w}}}_{{\bf{i}}}]-\frac{n}{2}\,\mathrm{log}\,|{\boldsymbol{\psi }}|-\frac{1}{2}\sum _{i\mathrm{=1}}^{n}{{{\bf{w}}}_{{\bf{i}}}}^{T}{{\boldsymbol{\psi }}}^{-1}{{\bf{w}}}_{{\bf{i}}}\\ =-\frac{1}{2}\sum _{i\mathrm{=1}}^{n}E[{\rm{tr}}({\boldsymbol{\Lambda }}{{\boldsymbol{\psi }}}^{-1}{{\boldsymbol{\Lambda }}}^{T}{{\bf{v}}}_{{\bf{i}}}{{{\bf{v}}}_{{\bf{i}}}}^{T})|{{\bf{w}}}_{{\bf{i}}}]+\sum _{i\mathrm{=1}}^{n}E{({{\bf{v}}}_{{\bf{i}}}|{{\bf{w}}}_{{\bf{i}}})}^{T}\Lambda {{\boldsymbol{\psi }}}^{-1}{{\bf{w}}}_{{\bf{i}}}-\frac{n}{2}\,\mathrm{log}\,|{\boldsymbol{\psi }}|-\frac{1}{2}\sum _{i\mathrm{=1}}^{n}{{{\bf{w}}}_{{\bf{i}}}}^{T}{{\boldsymbol{\psi }}}^{-1}{{\bf{w}}}_{{\bf{i}}}\mathrm{.}\end{array}$$

In the M step, we maximize the above expectation. To do so, we take derivatives of the log-likelihood function with respect to ${{\boldsymbol{\Lambda }}}_{x}$, ${{\boldsymbol{\Lambda }}}_{y}$ and ${{\boldsymbol{\Lambda }}}_{u}$, and obtain

8 $$\frac{\partial E\,\mathrm{log}\,l}{\partial {{\boldsymbol{\Lambda }}}_{x}}=\sum _{i\mathrm{=1}}^{n}{{\boldsymbol{\psi }}}_{x}^{-1}{{\boldsymbol{\Lambda }}}_{x}^{T}E({{\bf{z}}}_{{\bf{i}}}{{{\bf{z}}}_{{\bf{i}}}}^{T}|{{\bf{w}}}_{{\bf{i}}})-\sum _{i\mathrm{=1}}^{n}{{\boldsymbol{\psi }}}_{x}^{-1}{{\bf{x}}}_{{\bf{i}}}E{({{\bf{z}}}_{{\bf{i}}}|{{\bf{w}}}_{{\bf{i}}})}^{T},$$

9 $$\frac{\partial E\,\mathrm{log}\,l}{\partial {{\boldsymbol{\Lambda }}}_{y}}=\sum _{i\mathrm{=1}}^{n}{{\boldsymbol{\psi }}}_{y}^{-1}{{\boldsymbol{\Lambda }}}_{y}^{T}E({{\bf{z}}}_{{\bf{i}}}{{{\bf{z}}}_{{\bf{i}}}}^{T}|{{\bf{w}}}_{{\bf{i}}})+\sum _{i\mathrm{=1}}^{n}{{\boldsymbol{\psi }}}_{y}^{-1}{{\boldsymbol{\Lambda }}}_{u}^{T}E({{\bf{u}}}_{{\bf{i}}}{{{\bf{z}}}_{{\bf{i}}}}^{T}|{{\bf{w}}}_{{\bf{i}}})-\sum _{i\mathrm{=1}}^{n}{{\boldsymbol{\psi }}}_{y}^{-1}{{\bf{y}}}_{{\bf{i}}}E{({{\bf{z}}}_{{\bf{i}}}|{{\bf{w}}}_{{\bf{i}}})}^{T},$$

10 $$\frac{\partial E\,\mathrm{log}\,l}{\partial {{\boldsymbol{\Lambda }}}_{u}}=\sum _{i\mathrm{=1}}^{n}{{\boldsymbol{\psi }}}_{y}^{-1}{{\boldsymbol{\Lambda }}}_{u}^{T}E({{\bf{u}}}_{{\bf{i}}}{{{\bf{u}}}_{{\bf{i}}}}^{T}|{{\bf{w}}}_{{\bf{i}}})+\sum _{i\mathrm{=1}}^{n}{{\boldsymbol{\psi }}}_{y}^{-1}{{\boldsymbol{\Lambda }}}_{y}^{T}E({{\bf{z}}}_{{\bf{i}}}{{{\bf{u}}}_{{\bf{i}}}}^{T}|{{\bf{w}}}_{{\bf{i}}})-\sum _{i\mathrm{=1}}^{n}{{\boldsymbol{\psi }}}_{y}^{-1}{{\bf{y}}}_{{\bf{i}}}E{({{\bf{u}}}_{{\bf{i}}}|{{\bf{w}}}_{{\bf{i}}})}^{T},$$

where the conditional expectations are

11 $$E({{\bf{v}}}_{{\bf{i}}}|{{\bf{w}}}_{{\bf{i}}})={\boldsymbol{\Lambda }}{({\boldsymbol{\psi }}+{{\boldsymbol{\Lambda }}}^{T}{\boldsymbol{\Lambda }})}^{-1}{{\bf{w}}}_{{\bf{i}}},$$

12 $${\rm{Var}}({{\bf{v}}}_{{\bf{i}}}|{{\bf{w}}}_{{\bf{i}}})={\bf{I}}-{\boldsymbol{\Lambda }}{({\boldsymbol{\psi }}+{{\boldsymbol{\Lambda }}}^{T}{\boldsymbol{\Lambda }})}^{-1}{{\boldsymbol{\Lambda }}}^{T}$$

13 $$E({{\bf{v}}}_{{\bf{i}}}{{{\bf{v}}}_{{\bf{i}}}}^{T}|{{\bf{w}}}_{{\bf{i}}})={\rm{Var}}({{\bf{v}}}_{{\bf{i}}}|{{\bf{w}}}_{{\bf{i}}})+E({{\bf{v}}}_{{\bf{i}}}|{{\bf{w}}}_{{\bf{i}}})E{({{\bf{v}}}_{{\bf{i}}}|{{\bf{w}}}_{{\bf{i}}})}^{T}\mathrm{.}$$

The above equations form the basis of our EM algorithms.

## References

[1] Usoskin D (2015). Unbiased classification of sensory neuron types by large-scale single-cell rna sequencing. Nat Neurosci.

[2] Zeisel A (2015). Cell types in the mouse cortex and hippocampus revealed by single-cell rna-seq. Science.

[3] Jaitin DA (2014). Massively parallel single-cell rna-seq for marker-free decomposition of tissues into cell types. Science.

[4] Macosko EZ (2015). Highly parallel genome-wide expression profiling of individual cells using nanoliter droplets. Cell.

[5] Treutlein B (2014). Reconstructing lineage hierarchies of the distal lung epithelium using single-cell rna-seq. Nature.

[6] Tang F (2010). Tracing the derivation of embryonic stem cells from the inner cell mass by single-cell rna-seq analysis. Cell Stem Cell.

[7] Durruthy-Durruthy R (2014). Reconstruction of the mouse otocyst and early neuroblast lineage at single-cell resolution. Cell.

[8] Xue Z (2013). Genetic programs in human and mouse early embryos revealed by single-cell rna sequencing. Nature.

[9] Achim K (2015). High-throughput spatial mapping of single-cell rna-seq data to tissue of origin. Nat Biotechnol.

[10] Satija R, Farrell JA, Gennert D, Schier AF, Regev A (2015). Spatial reconstruction of single-cell gene expression data. Nat Biotechnol.

[11] Shalek, A. K. *et al*. Single-cell rna-seq reveals dynamic paracrine control of cellular variation. *Nature* **510**, 363−+; 10.1038/nature13437 (2014). 10.1038/nature13437 PMC4193940 24919153

[12] Kim KT (2015). Single-cell mrna sequencing identifies subclonal heterogeneity in anti-cancer drug responses of lung adenocarcinoma cells. Genome Biol.

[13] Lee MC (2014). Single-cell analyses of transcriptional heterogeneity during drug tolerance transition in cancer cells by rna sequencing. Proc Natl Acad Sci USA.

[14] Borel C (2015). Biased allelic expression in human primary fibroblast single cells. Am J Hum Genet.

[15] Deng Q, Ramskold D, Reinius B, Sandberg R (2014). Single-cell rna-seq reveals dynamic, random monoallelic gene expression in mammalian cells. Science.

[16] Stegle O, Teichmann SA, Marioni JC (2015). Computational and analytical challenges in single-cell transcriptomics. Nat Rev Genet.

[17] Vallejos CA, Marioni JC, Richardson S (2015). Basics: Bayesian analysis of single-cell sequencing data. PLoS Comput Biol.

[18] Kharchenko PV, Silberstein L, Scadden DT (2014). Bayesian approach to single-cell differential expression analysis. Nature Methods.

[19] Lun ATL, Bach K, Marioni JC (2016). Pooling across cells to normalize single-cell rna sequencing data with many zero counts. Genome Biology.

[20] Kumar N, Singh A, Kulkarni RV (2015). Transcriptional bursting in gene expression: Analytical results for general stochastic models. PLoS Computational Biology.

[21] Brennecke P (2013). Accounting for technical noise in single-cell rna-seq experiments. Nature Methods.

[22] Grun D, Kester L, van Oudenaarden A (2014). Validation of noise models for single-cell transcriptomics. Nat Methods.

[23] Kim JK, Kolodziejczyk AA, Illicic T, Teichmann SA, Marioni JC (2015). Characterizing noise structure in single-cell rna-seq distinguishes genuine from technical stochastic allelic expression. Nat Commun.

[24] Finak G (2015). Mast: a flexible statistical framework for assessing transcriptional changes and characterizing heterogeneity in single-cell rna sequencing data. Genome Biol.

[25] Reinius B, Sandberg R (2015). Random monoallelic expression of autosomal genes: stochastic transcription and allele-level regulation. Nat Rev Genet.

[26] Islam S (2014). Quantitative single-cell rna-seq with unique molecular identifiers. Nat Methods.

[27] Johnson WE, Li C, Rabinovic A (2007). Adjusting batch effects in microarray expression data using empirical bayes methods. Biostatistics.

[28] Walker WL, Liao IH, Donald L. Gilbert KSPCEMLL, Brenda W, Sharp FR (2008). Empirical bayes accomodation of batch-effects in microarray data using identical replicate reference samples: application to rna expression profiling of blood from duchenne muscular dystrophy patients. BMC Genomics.

[29] Risso D, Ngai J, Speed TP, Dudoit S (2014). Normalization of rna-seq data using factor analysis of control genes or samples. Nat Biotechnol.

[30] Leek JT, Storey JD (2007). Capturing heterogeneity in gene expression studies by surrogate variable analysis. PLoS Genet.

[31] Pickrell JK (2010). Understanding mechanisms underlying human gene expression variation with rna sequencing. Nature.

[32] Stegle O, Parts L, Durbin R, Winn J (2010). A bayesian framework to account for complex non-genetic factors in gene expression levels greatly increases power in eqtl studies. PLoS Comput Biol.

[33] Jiang L (2011). Synthetic spike-in standards for rna-seq experiments. Genome Res.

[34] Kang HM, Ye C, Eskin E (2008). Accurate discovery of expression quantitative trait loci under confounding from spurious and genuine regulatory hotspots. Genetics.

[35] Listgarten J, Kadie C, Schadt EE, Heckerman D (2010). Correction for hidden confounders in the genetic analysis of gene expression. Proc Natl Acad Sci USA.

[36] Jacob L, Gagnon-Bartsch JA, Speed TP (2015). Correcting gene expression data when neither the unwanted variation nor the factor of interest are observed. Biostatistics.

[37] Buettner F (2015). Computational analysis of cell-to-cell heterogeneity in single-cell rna-sequencing data reveals hidden subpopulations of cells. Nat Biotechnol.

[38] Sun Y, Zhang NR, Owen AB (2012). Multiple hypothesis testing adjusted for latent variables, with an application to the agemap gene expression data. Annals of Applied Statistics.

[39] Yang C, Wang L, Zhang S, Zhao H (2013). Accounting for non-genetic factors by low-rank representation and sparse regression for eqtl mapping. Bioinformatics.

[40] Gagnon-Bartsch JA, Speed TP (2012). Using control genes to correct for unwanted variation in microarray data. Biostatistics.

[41] Gagnon-Bartsch, J. A., Jacob, L. & Speed, T. P. Removing unwanted variation from high dimensional data with negative controls. Tech. Rep. (2013).

[42] Carvalho CM (2008). High-dimensional sparse factor modeling: Applications in gene expression genomics. Journal of the American Statistical Association.

[43] Pournara I, Wernisch L (2007). Factor analysis for gene regulatory networks and transcription factor activity profiles. BMC Bioinformatics.

[44] Lucas JE, Kung HN, Chi JT (2010). Latent factor analysis to discover pathway-associated putative segmental aneuploidies in human cancers. PLoS Comput Biol.

[45] Blum Y, Le Mignon G, Lagarrigue S, Causeur D (2010). A factor model to analyze heterogeneity in gene expression. BMC Bioinformatics.

[46] Parts L, Stegle O, Winn J, Durbin R (2011). Joint genetic analysis of gene expression data with inferred cellular phenotypes. PLoS Genet.

[47] Zhou X, Stephens M (2012). Genome-wide efficient mixed-model analysis for association studies. Nat Genet.

[48] Zhou X, Stephens M (2014). Efficient multivariate linear mixed model algorithms for genome-wide association studies. Nat Methods.

[49] Kiselev, V. Y. *et al*. Sc3: consensus clustering of single-cell rna-seq data. *Nature Methods* in press; (2017). 10.1038/nmeth.4236 PMC5410170 28346451

[50] Tung, J., Zhou, X., Alberts, S. C., Stephens, M. & Gilad, Y. The genetic architecture of gene expression levels in wild baboons. *Elife* **4**; 10.7554/eLife.04729 (2015). 10.7554/eLife.04729 PMC4383332 25714927

[51] Lea AJ, Tung J, Zhou X (2015). A flexible, efficient binomial mixed model for identifying differential dna methylation in bisulfite sequencing data. PLoS Genet.

[52] West M (2003). Bayesian factor regression models in the “large p, small n” paradigm. Bayesian Statistics.

[53] McDavid A, Finak G, Gottardo R (2016). The contribution of cell cycle to heterogeneity in single-cell rna-seq data. Nature Biotechnology.

[54] Marco E (2014). Bifurcation analysis of single-cell gene expression data reveals epigenetic landscape. Proc Natl Acad Sci USA.

[55] Grün D (2015). Single-cell messenger RNA sequencing reveals rare intestinal cell types. Nature.

[56] Xu C, Su Z (2015). Identification of cell types from single-cell transcriptomes using a novel clustering method. Bioinformatics.

[57] Pierson E, Yau C (2015). ZIFA: Dimensionality reduction for zero-inflated single-cell gene expression analysis. Genome Biology.

[58] Law CW, Chen Y, Shi W, Smyth G (2014). K. voom: Precision weights unlock linear model analysis tools for rna-seq read counts. Genome Biol.

[59] Ritchie ME (2015). limma powers differential expression analyses for rna-sequencing and microarray studies. Nucleic Acids Res.

[60] Soneson C, Delorenzi M (2013). A comparison of methods for differential expression analysis of rna-seq data. BMC Bioinformatics.

[61] Seyednasrollah F, Laiho A, Elo LL (2015). Comparison of software packages for detecting differential expression in rna-seq studies. Brief Bioinform.

[62] Lappalainen T (2013). Transcriptome and genome sequencing uncovers functional variation in humans. Nature.

[63] Battle A (2014). Characterizing the genetic basis of transcriptome diversity through rna-sequencing of 922 individuals. Genome Res.

[64] Montgomery SB (2010). Transcriptome genetics using second generation sequencing in a caucasian population. Nature.

[65] Lee S, Chugh PE, Shen H, Eberle R, Dittmer DP (2013). Poisson factor models with applications to non-normalized microrna profiling. Bioinformatics.

[66] Zhou M, Hannah L, Dunson D, Carin L (2012). Beta-negative binomial process and poisson factor analysis. Artificial Intelligence and Statistics.

